# Mitogenomic analysis of diversity of key whitefly pests in Kenya and its implication to their sustainable management

**DOI:** 10.1038/s41598-021-85902-2

**Published:** 2021-03-18

**Authors:** Fathiya M. Khamis, Fidelis L. O. Ombura, Inusa J. Ajene, Komivi S. Akutse, Sevgan Subramanian, Samira A. Mohamed, Thomas Dubois, Chrysantus M. Tanga, Sunday Ekesi

**Affiliations:** Plant Health Theme, International Centre of Insect Physiology and Ecology (icipe), P.O. Box 30772-00100, Nairobi, Kenya

**Keywords:** Ecology, Molecular biology

## Abstract

Whiteflies (Hemiptera: Aleyrodidae) are devastating agricultural pests of economic importance vectoring pathogenic plant viruses. Knowledge on their diversity and distribution in Kenya is scanty, limiting development of effective sustainable management strategies. The present study is aimed at identifying whitefly pest species present in Kenya across different agroecological zones and establish predictive models for the most abundant species in Africa. Whiteflies were sampled in Kenya from key crops known to be severely infested and identified using 16S rRNA markers and complete mitochondrial genomes. Four whitefly species were identified: *Aleyrodes proletella*, *Aleurodicus dispersus*, *Bemisia afer* and *Trialeurodes* *vaporariorum*, the latter being the most dominant species across all the agroecology. The assembly of complete mitogenomes and comparative analysis of all 13 protein coding genes confirmed the identities of the four species. Furthermore, prediction spatial models indicated high climatic suitability of *T. vaporariorum* in Africa, Europe, Central America, parts of Southern America, parts of Australia, New Zealand and Asia. Consequently, our findings provide information to guide biosecurity agencies on protocols to be adopted for precise identification of pest whitefly species in Kenya to serve as an early warning tool against *T. vaporariorum* invasion into unaffected areas and guide appropriate decision-making on their management.

## Introduction

Whiteflies (Hemiptera: Aleyrodidae) are devastating agronomic pests of economic importance in warmer parts of the globe as well as temperate regions^1^. Direct damage by whiteflies is caused by the piercing-sucking behaviour of both adults and nymphs. Infestations by whiteflies cause over 50% crop yield reduction^2–4^ while as early as 1989 in Florida, feeding by immature stages was estimated to have caused a loss of $25 million in tomato production due to irregular ripening^5,6^. Honeydew excreted by whiteflies on the crop foliage favours the growth of sooty mold, caused by group of Ascomycete fungi, which reduces photosynthetic capacity of the leaves. The sooty mold also reduces the aesthetic quality of crops and consequently their marketability^7^. Most importantly, whiteflies are major vectors of pathogenic viruses in a wide range of crops.

The most damaging and widespread whitefly species are the cotton or sweet potato whitefly, *Bemisia tabaci* (Gennadius) and the greenhouse whitefly, *Trialeurodes vaporariorum* (Westwood)^8,9^. *Bemisia* *tabaci*, a cryptic species complex with a world-wide distribution, is the most significant pest^10,11^ attacking most crops of Brassicaceae, Solanaceae, Malvaceae, Asteraceae, Fabaceae, Apocynaceae, Chenopodiaceae, Rutaceae, Cucurbitaceae, Euphorbiaceae, Balsaminaceae, Moraceae, Lamiaceae, Piperaceae, and Gesneriaceae families^12^. The pest has been associated with vectoring more than 100 viruses^13–15^ most notably geminiviruses in the genus Begomovirus (Geminiviridae)^16^, cassava mosaic begomoviruses (CMBs) and cassava brown streak viruses (CBSVs)^17,18^. The second most important whitefly species is *T. vaporariorum,* a significant pest of vegetable and ornamental crops worldwide^19^. The species is highly polyphagous and thrives on over 300 species of host plants including; tomato (*Solanum lycopersicum*), beans (*Phaseolus vulgaris*), eggplant (*Solanum melongena*), sweet pepper (*Capsicum annuum*), potato (*Solanum tuberosum)*, tobacco (*Nicotiana tabacum*), cabbage (*Brassica oleracea* var. *capitata)*, cucumber (*Cucumis sativus*), squash (*Curcubita* spp.), cotton (*Gossypium* spp*.*), hibiscus (*Hibiscus rosa-sinensis*), poinsettia (*Euphorbia pulcherrima*) and begonia (*Begonia* spp*.*) as well as several weed species^20^. *Trialeurodes* *vaporariorum* has been reported to transmit several viruses within the genus *Crinivirus* such as *Tomato infectious chlorosis virus* (TICV), *Tomato chlorosis virus* (ToCV), *Strawberry pallidosis associated virus* (SPaV), *Potato yellow vein virus* (PYDV) and *Beet pseudo yellows* *virus* (BPYV)^9^. *Trialeurodes* *vaporariorum* also vectors several bacterial species and has been reported to spread *Polyphagotarsonemus latus* mites^19,20^.

Despite the economic significance of whiteflies on vegetables in Africa, most surveys and research has mainly focused on *B.* *tabaci* on cassava^16, 21–25^. Previous studies conducted between 1997 and 1998 in Kenya documented *B. tabaci* as the main pest and vector of viruses on tomato^26^. Furthermore, identification of whiteflies to the species level is based almost entirely on the morphological characters of the pupal stage^27^. However, routine microscopic characterization of pupae is time-consuming, and requires a high level of training and expertise. In addition, pupae are notorious for displaying high intraspecific variation induced by host plant and environmental factors and thus limit accurate identification^28^. Adult specimens are identified occasionally, although they have very few diagnostic characters that can only be detected using a scanning electron microscope^28^. Morphological identification of whiteflies is also hampered by the presence of damaged specimens and cryptic species such as the numerous biotypes of *B. tabaci*^29,30^. Molecular taxonomic approaches represent a valuable addition to morphological taxonomy and have proven useful in rapid identification of whitefly species and discrimination of biotypes within the *B. tabaci* species complex^28–33^. Furthermore, the use of high throughput long read sequencing aids in the resolution of the taxonomic composition of metagenomes up to the species level^34^. Therefore, accurate identification and appropriate monitoring of whiteflies and their biotypes in Kenya is a prerequisite for their efficient management, specifically for targeting natural enemies.

**Figure 1:**
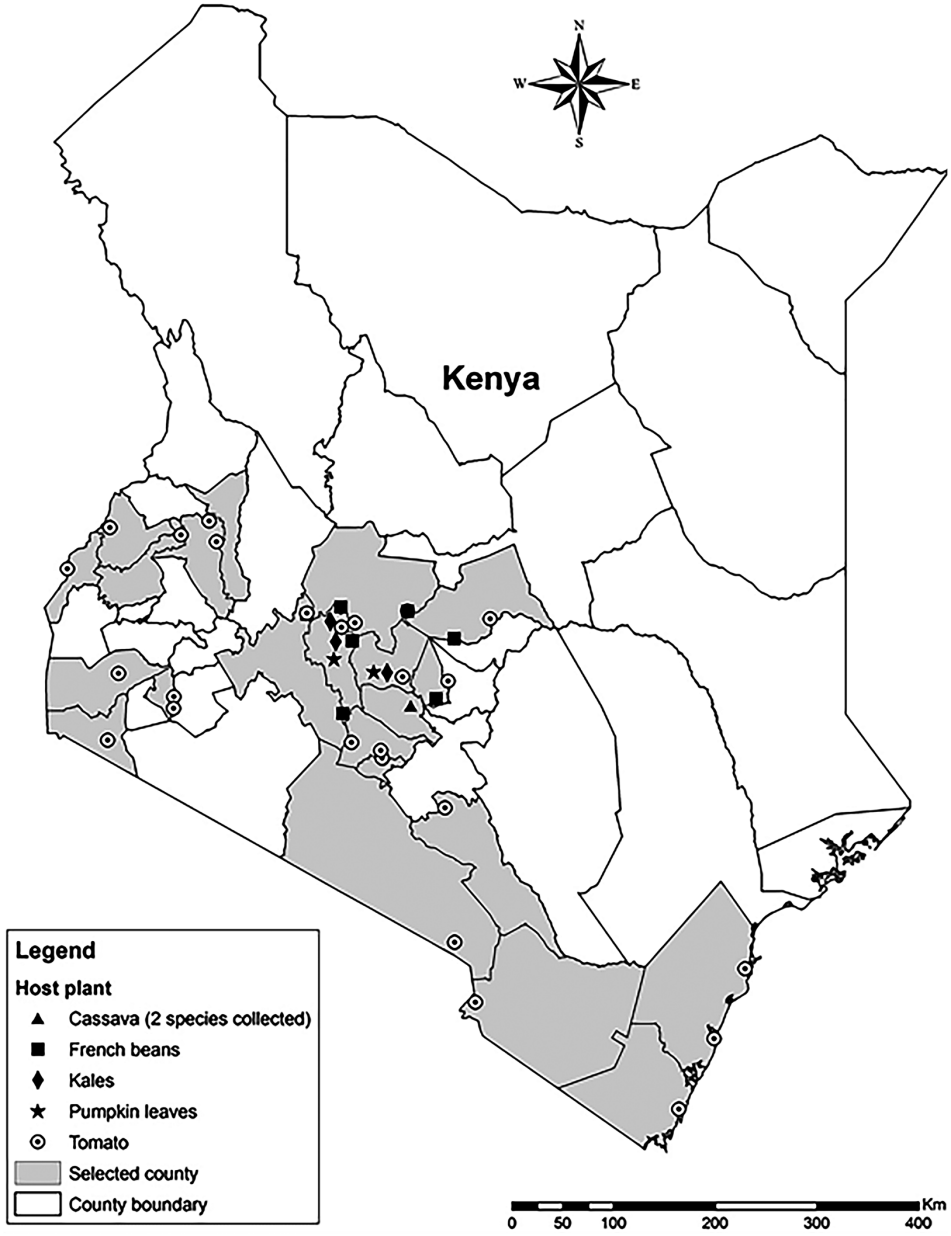
Distribution map of *Trialeurodes vaporariorum* in Kenya.

In this study, a survey was conducted (Fig. 1) to document the identity, distribution and diversity of whitefly species on tomato, French bean, kales, butternut, pumpkin and cassava in Kenya using the mitochondrial genome. Furthermore, the current and future potential distribution of the most abundant species, was mapped to provide insight into the current and potential future distribution of the species.

## Results

### Whitefly species identification and diversity as per the various host plants

**Figure 2:**
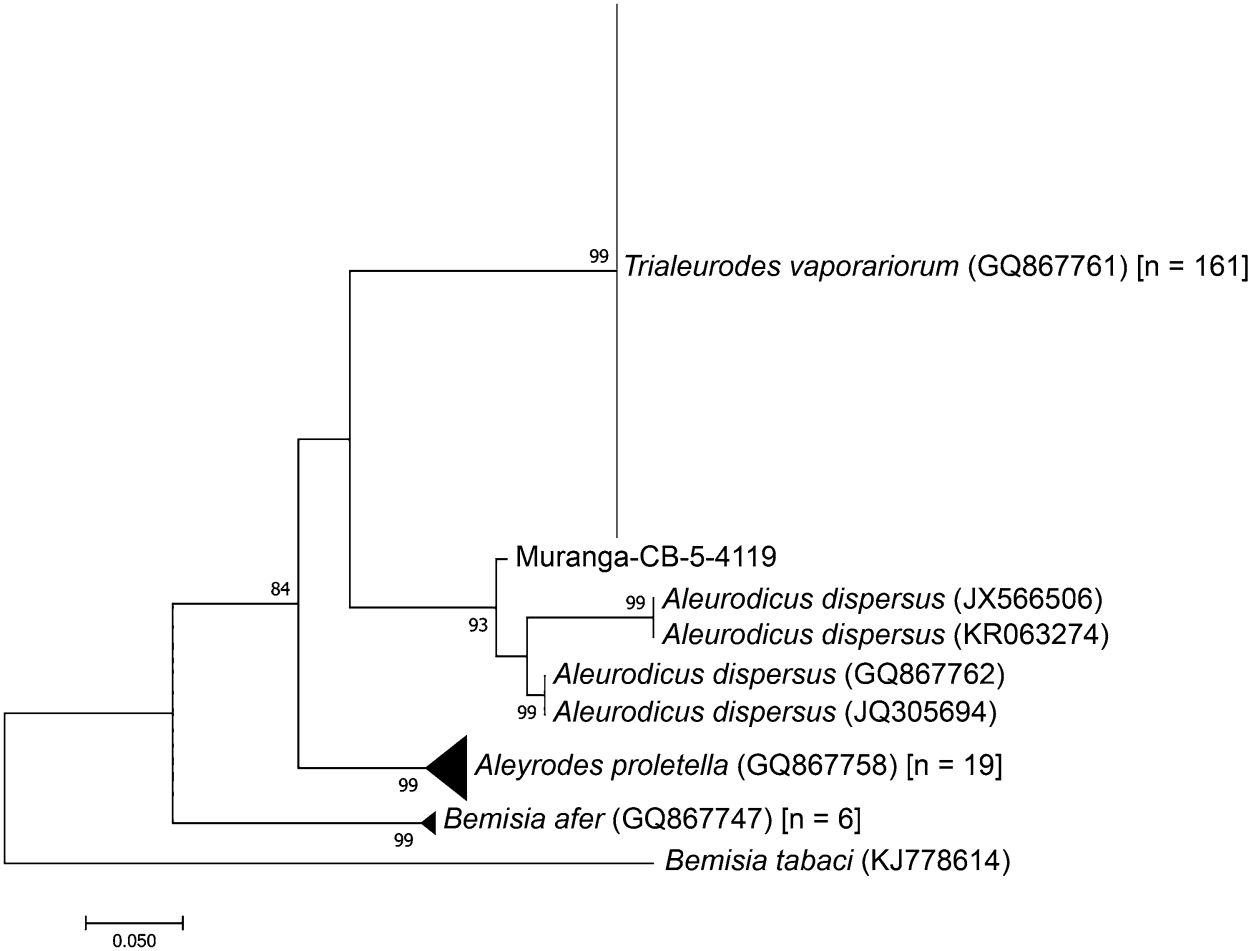
Maximum-likelihood tree based on a 650 bp alignment of 188 sequences of the 16S ribosomal RNA (*rrna)* gene using the 4119/4118 markers from whitefly samples collected from Kenya and reference whitefly sequences available in GenBank (n = 5), with *Aphis gossypii* as an outgroup. The number of sequences obtained in this study are indicated in square brackets. Branchsupport was based on 1000 bootstrap replicates.

**Figure 3:**
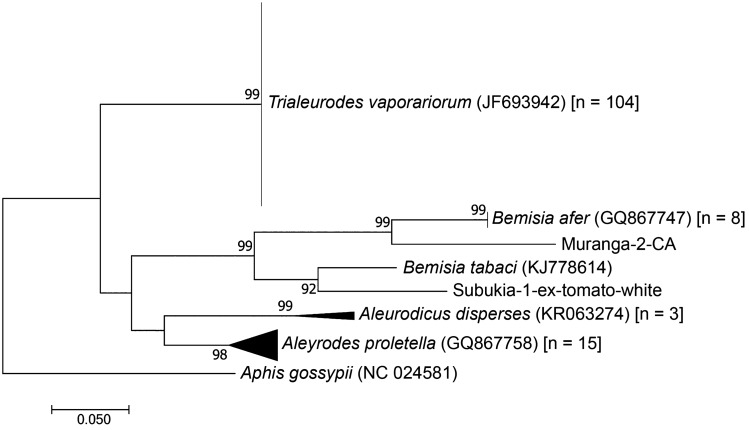
Maximum-likelihood tree based on a 650 bp alignment of 132 sequences of the 16S ribosomal RNA (*rrna)* gene using the whitefly specific from whitefly samples collected from Kenya and reference whitefly sequences available in GenBank (n = 5), with *Aphis gossypii* as an outgroup. The number of sequences obtained in this study is indicated in square brackets. Branchsupport was based on 1000 bootstrap replicates.

The phylogenetic reconstruction unraveled the various species/sub-types of whiteflies commonly found on tomato, French beans, kales, pumpkin and cassava. A total of 188 sequences were successfully obtained using the 4119/4118 markers while 132 sequences were recovered using the whitefly specific primers. The most abundant whiteflies collected from tomato and kales belonged to the species *T. vaporariorum* and *Bemisia afer*. On French beans, only *T. vaporariorum* were identified, while *Aleurodicus dispersus* and *B. afer* were more specific to cassava. All the identity levels were ≥ 99% as queried in BLAST at GenBank. From both sets of analysis, there were ≥ 3 main clusters. Using the 4119/4118 markers, the Kenyan samples clustered into four clades as *A. proletella*, *A. dispersus*, *B. afer* and *T. vaporariorum* (Fig. 2). Similarly, the whitefly specific markers discriminated the samples into five main clades i.e. *A. dispersus* (from cassava), *A. proletella* (from kale), *B. afer* (from cassava and French beans), *B. tabaci* (from tomato), and *T. vaporariorum* (from pumpkin, tomato, butternut, kales and French beans) (Fig. 3).

### The complete mitogenome sequences of whiteflies from Kenya

In the present study, 1.7 × 10^7^, 1.9 × 10^7^, 1.9 × 10^7^ and 1.8 × 10^7^ reads were generated for *A. disperses*, *B. afer*, *T. vaporariorum* and *A. proletella* samples, respectively, from MiSeq sequencing. A de novo assembly of the *A. proletella* sample generated 812,145 contigs (≥ 1000 bp). One contig of 14,563 bp was identified by BLASTn. For the reference-based assembly of the whitefly samples, 39,900, 145,385 and 164,997 reads were generated for *A. dispersus*, *B. afer* and *T. vaporariorum*, respectively. Referenced to KR063274, NC_024056 and KY426015, contigs of 17,327 bp, 17,664 bp and 16,580 bp were identified for *A. dispersus*, *B. afer* and *T. vaporariorum*, respectively.

Sequence annotation showed that all the mitogenomes comprised of the entire set of 37 genes typical of metazoan mitochondrial genomes, including 13 protein-coding genes (PCGs), 22 transfer RNA (tRNA) genes and two ribosomal RNA (rRNA) genes. All the three complete mitogenomes had the same gene order, gene direction and start/stop codons of each PCGs as that of the reference sequences (Supplementary Table 6a to 6c).

**Figure 4:**
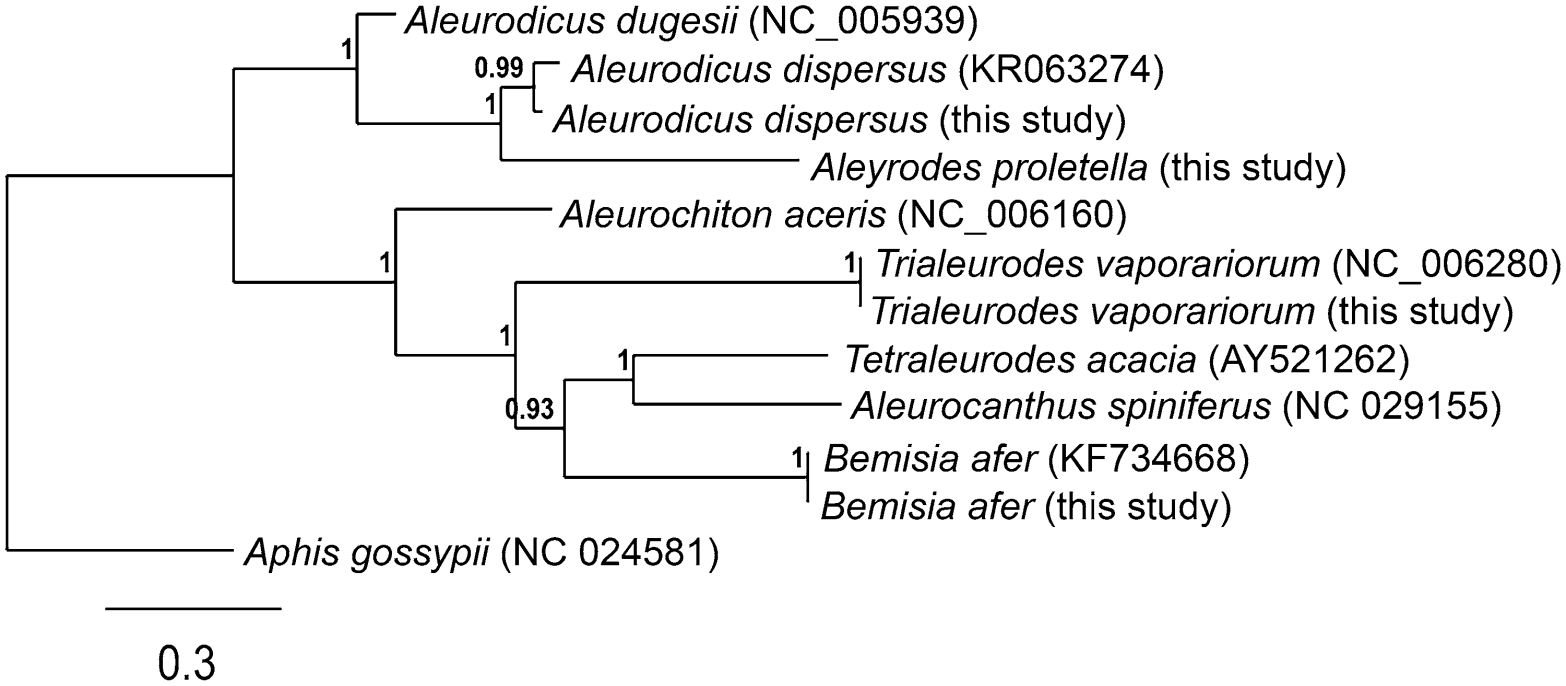
Maximum-likelihood tree of full mitogenomes (13 protein coding genes) based on complete mitogenome sequences excluding tRNA, rRNA and control regions of *Aleurodicus dispersus*, *Aleyrodes proletella*, *Bemisia afer and Trialeurodes vaporariorum* from Kenya including *Bemisia tabaci* mitogenome available in GenBank, with *Aphis gossypii* mitogenome as an outgroup. Branchsupport was based on 1000 bootstrap replicates. Numbers in the brackets represent the GenBank accession numbers.

The pairwise similarity between the samples from this study and the GenBank reference samples showed 94.01% between *A. dispersus* and the reference mitogenome, 99.56% between *B. afer* from this study and the reference mitogenome and 94.52% between *T. vaporariorum* from this study and the reference mitogenome (Supplementary Table 7). The maximum likelihood tree of the 13 PCG’s from the samples in this study combined with representative sequences, showed a paraphyletic relationship between the *Aleurodicus* genus and the other whitefly genera (Fig. 4).

**Figure 5:**
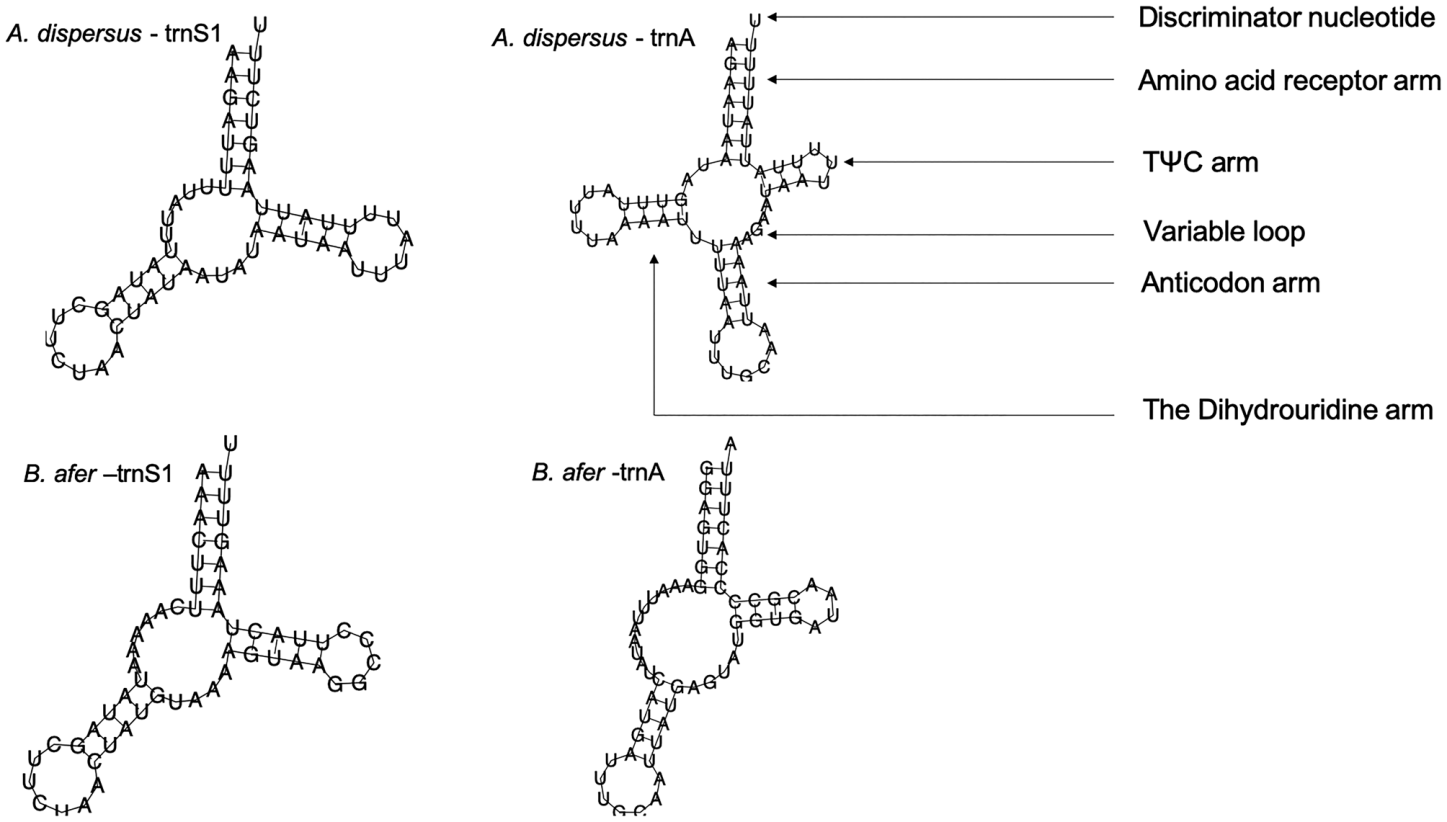
Transfer RNA secondary structure of trnA and trnS1 in *Aleurodicus dispersus* and *Bemisia afer* as predicted by MITOS showing presence and absence of dihydrouridine arm.

The combined length of all tRNA genes was 1309 bp, 1459 bp and 1422 bp for *A. dispersus*, *B. afer* and *T. vaporariorum*, respectively; with individual genes ranging from 57 to 80 bp for *A. dispersus*, 57 to 83 bp for *B. afer* and 55 to 70 bp for *T. vaporariorum*. Analysis of the tRNA secondary structure showed that all the tRNAs in *A. dispersus* (Supplementary Fig. 1) and *T. vaporariorum* (Supplementary Fig. 2) had the typical clover leaf structure except for trnS1 and trnS2, which lack the dihydrouridine (DHU) arm (Fig. 5). Nineteen of the tRNAs in *B. afer* (Supplementary Fig. 3) showed the typical clover leaf structure, except for trnA, trnS1 and trnS2 which lacked the DHU arm (Fig. 5).

## Potential distribution of *Trialeurodes vaporariorum* under current and future scenarios

**Figure 6:**
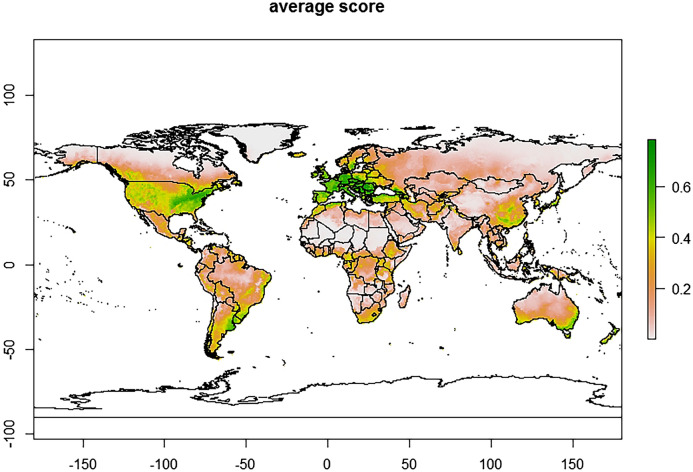
Maxent and BIOCLIM average model plots depicting current establishment of *Trialeurodes vaporariorum* with an establishment risk index (ERI) > 0.6 in Europe, Central America, parts of Southern America (Brazil, Uruguay and Argentina), parts of Australia, New Zealand and Asia (China and Japan).

**Figure 7:**
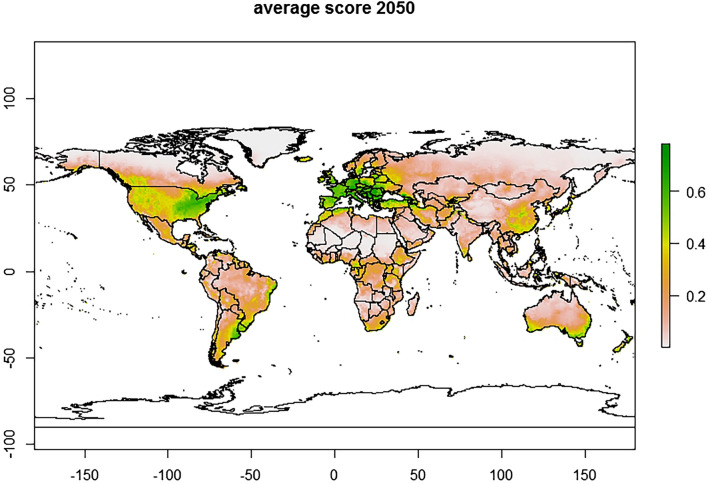
Maxent and BIOCLIM average model plots depicting future changes in the establishment of *Trialeurodes vaporariorum.* Prediction is up to year 2050 showing increase in risk of *Trialeurodes vaporariorum* establishment (ERI > 0.4–0.6) probably due to the rising temperatures attributed to global warming.

**Figure 8:**
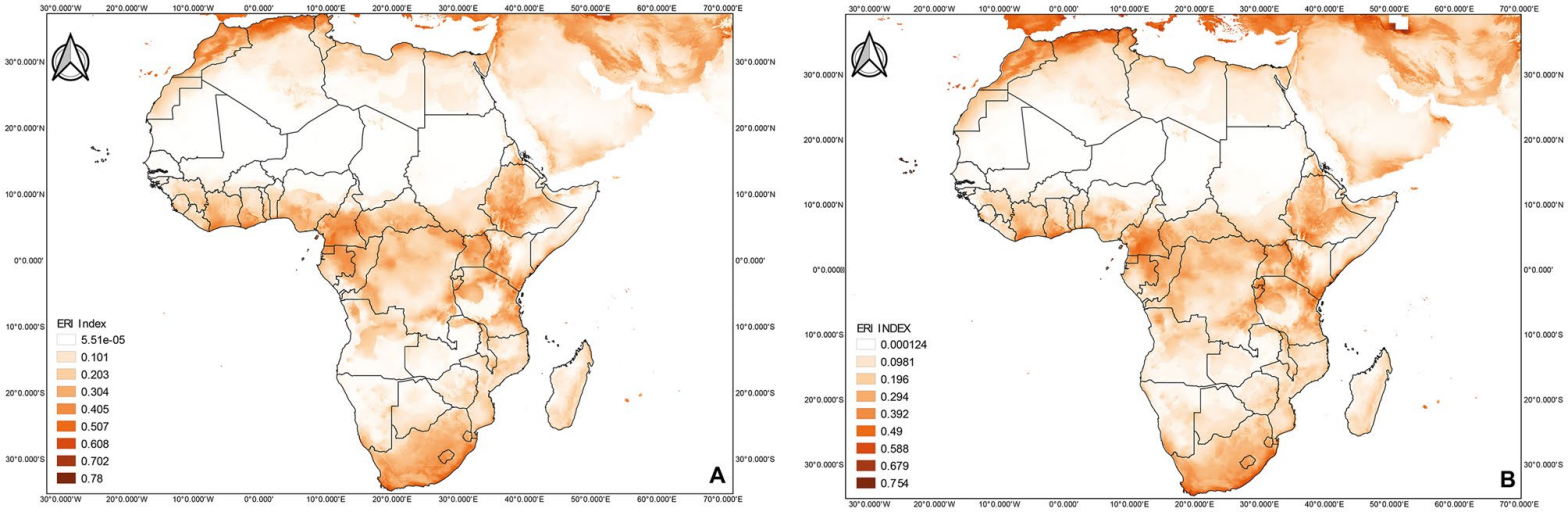
Maxent and BIOCLIM average model plots depicting African continent (**A**) current (2019) and (**B**) future (2050) potential establishment of *Trialeurodes vaporariorum.*

The predicted current global distribution of *T. vaporariorum* obtained from the MaxEnt model showed the highest likelihood of establishment in most European countries, the USA, southern Canada, Brazil, Uruguay, Argentina, Australia, New Zealand, Northern Iran, South eastern China and Japan (Fig. 6) with an establishment risk index (ERI) of > 0.6. The predicted future global distribution showed the risk of establishment of *T. vaporariorum* showed an increase under future climate scenario, with ERI ranging between > 0.4–0.6 (Fig. 7). The predicted current distribution of *T.* *vaporariorum* in Africa ranged between 0.101 and 0.78. The countries considered to be highly suitable for the pest establishment with an ERI of > 0.4, included Algeria, Cameroon, Ethiopia, Gabon, Kenya, Morocco, South Africa, Tanzania, and Uganda (Fig. 8a). Under the future climate scenario, the predicted distribution in Africa ranged between 0.0981 and 0.754 (Fig. 8b).

## Discussion and conclusion

In this study, we identified the various whitefly species present on a range of vegetable crops in Kenya and their species diversity. Subsequently we identified the most abundant whitefly species (*T. vaporariorum*) then modelled the potential current and future risk of establishment of *T. vaporariorum* to provide critical information on the global distribution and establishment of the species.

Frohlich^35^ demonstrated the importance of the mitochondrial cytochrome oxidase subunit I (COI) as a region for analysing nucleotide divergence between and within populations. However, we experienced low amplification efficiencies with the primers used in the study described by Folmer^36^, which is in tandem with the findings reported by Shatters^30^ who also observed low quality and suboptimal product quantities when using mtCOI primers; C1-J-2195 and L2-N-3014. Considering this, our study focused on using the whitefly 16S ribosomal subunit. In nature, insects are known to co-evolve depending on the prevailing biotic factors and most often they develop morphologically indistinct sibling species as observed in black flies^37^, mosquitoes^38^ among others. Whiteflies have shown similar attributes hence the rise to the numerous biotypes distributed in the world. However, according to the review done by De Barro^10^, the use of biotype in describing *B. tabaci* and other whitefly species was thought to be erroneous and misleading. We report on the characterization of four distinct whiteflies species found in various agro-ecological zones of Kenya with two of the described species co-existing on the same crop. To date, *B. tabaci* has at least 24 known morphologically indistinguishable species^39^. This possesses a real challenge in developing sustainable effective control approaches since these species differ in terms of their susceptibility to natural enemies’, host range, insecticide resistance, fecundity and vector competence for begomoviruses^40,41^.

From our study, we were able to detect four different species of whiteflies from the various host plants assayed in the country. Among these species, *T.* *vaporarium* is the most abundant in Kenya. This was mainly found on both tomato and French bean crops while the traditionally known whitefly, *B. tabaci* was not detected, which is contrary to earlier findings in tomato agroecology of Kenya where it was reported as the dominant species collected^26^. Our sequences matched at ≥ 98% with *T. vaporarium* available at GenBank. This level of homology was confirmed via use of 3 different markers. The analysis of the complete mitochondrial genomes of the four species also confirmed the homology of each species to its reference mitogenome. When the reference sequences from GenBank were included in the phylogenetic assays, it was noticed that the Kenyan whitefly samples completely diverged from the *B. tabaci* complex. It was also observed that none of the samples mapped to *B. tabaci* in the assembly of the NGS sequences. We can therefore infer that though *B. tabaci* is a devastating pest, it could either have been displaced by *T. vaporarium* or out-competed from the Kenyan agroecological zones, or its current biotype population is low, and thus we may be faced with threats from other whiteflies species which were previously not considered to be economically important. Effects of indiscriminate chemical use over time cannot be understated, and hence we cannot rule out the possibility of near ‘almost complete’ wipe-out of *B. tabaci* in the Kenyan agro-ecological zones. In this study, *B. afer* (which is an out-group in the *B. tabaci* complex) and *A. dispersus* were found co-existing on the same cassava plant, a known host plant of *B. tabaci* whereas *A.* *proletella* which was identified on kales has been documented to prefer cabbage^42,43^ as a host plant.

The maximum likelihood analysis supported the separation of the Aleyrodidae into subfamilies Aleurodicinae and Aleyrodinae with *A*. *dugesii* and *A. dispersus* belonging to Aleurodicinae. This is consistent with the reports of Chen^44^, who also demonstrated that *A. dugessii* belonged to the subfamily Aleurodicinae. In the Aleyrodinae subfamily, *A*. *spiniferus* formed a clade with *T*. *acacia* while *B*. *tabaci* species grouped together with *B*. *afer*. These results are consistent with the morphological classification and phylogenetic studies of whiteflies^42–47^. The analysis showed a paraphyletic relationship among the Aleyroididae family. Monophyly was observed between *Bemisia* clade and the *Aleurocanthus spiniferus* and *Tetraleuroides acaciae* clades.

Gamarra^48^ used life table parameters to map out the distribution of *T. vaporariorum* and constructed the first risk atlas for Africa. The study predicted that agroecological zones with ERI ≥ 0.6 would have permanent/life-long establishment of this pest. This is in congruence with our findings whereby our models (Fig. 6) projected ERI > 0.6 in Europe, Central America, parts of Southern America (Brazil, Uruguay, and Argentina), parts of Australia, New Zealand, and Asia (China and Japan). In areas where open field farming is practiced, as evidence in the greater Asian and African continents, the ERI lessened to 0.2–0.4 (Fig. 6). Global temperature changes have been on the rise and this warming could have a varying effect on the establishment of *T. vaporariorum* (ERI > 0.4–0.6). The future prediction in this study (Fig. 7) shows that despite *T. vaporariorum* being native to Brazil or Mexico, other areas (Central America, Europe, parts of India and China) are at greater threat of the pest’s suitable and permanent establishment. In most instances, chemical control has been used exhaustively to curb the spread of this pest but with limited results and pesticide resistance development, hence this study emphasizes on the importance of a holistic approach for the management of whiteflies based on the use of biopesticides, parasitoids, mass trapping and some recommended pesticides. Furthermore, based on the observed model, the risks of *T. vaporariorum* establishment in Africa is predicted to decrease albeit slightly with an ERI difference of 0.0231 between the current and the future predictions. *Trialeurodes* *vaporariorum* is a temperate pest; therefore, although, the decrease in the potential establishment could be due to the inconsistencies in the climatic conditions especially rising temperatures attributed to global warming, the difference will probably not affect the long-term establishment of *T. vaporariorum* as the average ERI falls within the range of permanent establishment of the pest^48^. Here in this study, the detection of different whitefly species clearly indicates that the biological control agents must be well screened for host specificity, if meaningful control options are anticipated. The most abundant species collected was *T. vaporariorum*, and if the dispersal trajectory is maintained, the life-long establishment of *T. vaporariorum* would be global. Hence, *T. vaporariorum* will remain a potential risk and threat for food production in the high-risk regions if sustainable effective measures are not developed to suppress the pest dynamics.

## Methods

### Sampling

Whiteflies were sampled from various host plants; tomato (*Solanum lycopersicum*), butternut (*Cucurbita moschata*), French beans (*Phaseolus vulgaris*), kales (*Brassica oleracea*), pumpkin (*Curcubita moschata*) and cassava (*Manihot esculenta*) in farmer fields across different agro-ecological zones and altitude levels in Kenya from the year 2015–2019 (Supplementary Tables 1 and 2). No plant materials were collected from the field during the survey except insect samples. These insects were pests on several vegetables crops and were not endangered or protected species. No permission was required to sample these insects from the farmer fields since *icipe* operates under a Headquarters’ agreement with the Kenyan Government. During the survey, the insects were collected at random (from 5 plants per each row, at an interval of three plants, in a zig zag pattern) from the underside of the leaves using a mouth aspirator. About 30 adult flies were collected from each locality and immediately preserved in 95% ethanol before taken to the laboratory. The samples stored were at − 20 °C in the laboratory awaiting further processing. Global positioning system (GPS) coordinates were recorded for each sampled locality using Garmin eTrex20 instrument (GARMIN, Olathe, Kansas, USA) (Fig. 1).

### DNA extraction, PCR and sequencing

Each individual insect was surface sterilized using 3% NaOCl and rinsed three times with distilled water. Genomic DNA was extracted using the Isolate II genomic DNA Kit^49^ (Bioline, London, UK), following the manufacturer’s instructions. The purity and concentration of the resultant extracted DNA was determined using Nanodrop 2000/2000c Spectrophotometer (Thermo Fischer Scientific, Wilmington, USA) then stored at − 20 °C for used in downstream processes. Polymerase chain reaction (PCR) was done to amplify a portion of the 16S ribosomal RNA gene region using 2 sets of markers^50–52^ (Supplementary Table 3). The PCR was carried out in a total reaction volume of 20 µL containing 5X My *Taq* Reaction Buffer (5 mM dNTPs, 15 mM MgCl_2,_ stabilizers and enhancers) (Bioline, London, UK), 0.5 pmol µl^−1^ of each primer, 0.5 mM MgCl_2_, 0.0625 U µl^−1^ My *Taq* DNA polymerase and 15 ng µl^−1^ of DNA template. This reaction was set up in the Mastercycler gradient Nexus thermal cycler (Eppendorf, Hamburg, Germany). The following cycling conditions were used: initial denaturation for 2 min at 95 °C, followed by 40 cycles of 30 s at 95 °C, 40 s annealing and 1 min at 72 °C, then a final elongation step of 10 min at 72 °C. The target gene region was 650–700 base pairs. The amplified PCR products were resolved through a 1.2% agarose gel. The DNA bands on the gel were analyzed and documented using KETA GL imaging system trans-illuminator (Wealtec Corp, Meadowvale Way Sparks, Nevada, USA). Successively amplified products were excised and purified using Isolate II PCR and Gel Kit (Bioline) following the manufacturer’s instructions. The purified samples were shipped to Macrogen Inc Europe Laboratory, Amsterdam, the Netherlands, for bi-directional sequencing and the sequences were deposited in GenBank (Table 1). After identification using the afore-mentioned markers, NGS using Illumina MiSeq was carried out on DNA extracts from representative samples for the analysis of the mitochondrial genome of the samples.

**Table 1:** Summary of the assigned GenBank accessions of samples/sequences generated in this study.

Species	16S ribosomal RNA gene	Mitogenome
*Aleurodicus dispersus*	MW599223–MW599225, MW644551	SAMN14567375 (BioProject PRJNA624105)
*Aleyrodes proletella*	MW599226, MW599958–MW599972, MW644553–MW644566, MW644569–MW644572	*
*Bemisia afer*	MW599227–MW599234, MW646418–MW646419	SAMN14567251 (BioProject PRJNA624105)
*Bemisia tabaci*	MW599235, MW646417	*
*Trialeurodes vaporariorum*	MW603037–MW603179	SAMN14567119 (BioProject PRJNA624105)

*Partial mitogenome.

The successful sequences were assembled and edited using Geneious Version 8 (http://www.geneious.com)^53^ followed by multiple alignments done in Clustal X version 2.1^54^. For conclusive identification of the species, both similarity and phylogenetic analyses were carried out. Similarity searches were conducted by querying the consensus sequences via the Basic Local Alignment Search Tool (BLAST). The (BLAST) algorithm was used to identify regions of similarity between sequences from this study and publicly available sequences in the GenBank database (Supplementary Table 4). Using the clean assembled sequences, phylogenetic and molecular evolutionary analyses were conducted in MEGA X^55^. In order to infer the biotypes of the Kenyan whitefly collections, reference sequences were retrieved from GenBank and included in the analyses. These included *A. dispersus* (KR063274), *A. proletella* (GQ867758), *B. afer* (GQ867747), *B. tabaci* (KJ778614) and *T. vaporariorum* (GQ867761). Pairwise and multiple alignment of the sequences was done using Clustal W in MEGA. Evolutionary relationships between the various samples were inferred using the maximum likelihood algorithm method based on the Kimura 2-parameter model^56^ as implemented in MEGA X. The reliability of the trees was assessed using 1000 bootstrap replications.

### Complete mitogenome and phylogenetic analyses

Reference based assembly of the mitogenome sequences were carried out in Geneious Version 8 using the partial mitochondrial genome sequence of *A. dispersus* (KR063274) and complete mitochondrial genome sequence of *B. afer* (KF734668) and *T. vaporariorum* (NC_006280) as reference. De-novo assembly of mitogenome sequence for *A. proletella*, which did not have a partial or complete record in GenBank, was performed with SPAdes v.3.13.0. Contigs were identified by BLASTn v.2.2.30^57^. The mitogenome sequence was annotated by MITOS^58^ (the complete mitogenomes of *A. dispersus*, *B. afer* and *T. vaporariorum* have been uploaded to GenBank (Table 1).

Reference mitogenomes of *A. dispersus* (KR063274), *B. afer* (KF734668), *B. tabaci* (KJ778614) and *T. vaporariorum* (NC_006280) (Supplementary Table 5) were downloaded from the GenBank and assembled mitogenomes from this study were aligned using Clustal X. Thirteen Protein coding genes (PCGs) excluding the tRNA’s, rRNA’s and putative control regions were extracted and aligned with the reference mitogenomes using Clustal X. Maximum likelihood method was implemented in PhyML for the construction of the phylogenetic tree. The mitogenome of *Aphis gossypii* (NC_024581) was used as outgroup. Bootstrap analysis of 1000 replicates were used for nodal support.

### *Trialeurodes vaporariorum* spatial distribution modelling

Global occurrence data were obtained from CABI Invasive Species Compendium (https://www.cabi.org/ISC/datasheet/54660)^59^. Environmental predictors included 19 bioclimatic variables of 2.5 km spatial resolution for the current scenario using the baseline average [1950–2000]^60^, and for the future scenario using the Coupled Model Intercomparison Project, Phase 5 (CMIP) 2055 average [2041–2070]^61^. The environmental variables were obtained from the WorldClim database (http://www.worldclim.org/). The bioclimatic variables represent annual trends (e.g., mean annual temperature, annual precipitation), seasonality (e.g., annual range in temperature and precipitation) and extreme or limiting environmental factors (e.g., temperature of the coldest and warmest month, and precipitation of the wet and dry quarters)^62^. All these variables were created using the 'biovars' function in the R package ‘dismo’ version 1.1-4 and Maxent version 3.4.1 (R studio). The strongly correlated variables among all the bioclimatic variables were removed using the “findCorrelation” function in the ‘Caret’ package^63^ in R v3.4.1 and only the uncorrelated predictor variables [Annual mean temperature (Bio 1), Temperature annual range (Bio 2), Mean diurnal range (Bio 7), Mean temperature of warmest quarter (Bio 10), Precipitation of wettest month (Bio 13), Precipitation of driest month (Bio 14), Precipitation seasonality (Bio 15), Precipitation of warmest quarter (Bio 18) and Precipitation of coldest quarter (Bio 19)] were subsequently used. Presence locations for *T. vaporariorum* (occurrence data) were compared against all the pseudo-absence points that are available in the study area to avoid model overfitting of spatially clustered presence points and inability to predict spatially independent data. The model was trained using 75% of the presence data and validated using 25%. The model was run with 5000 iterations and > 10,000 background points for both current and future climate scenarios.

### Ethics approval

All handling and experiments were performed as per the procedures at the ICIPE Animal Rearing and Quarantine Unit as approved by the National Commission of Science, Technology and Innovations, Kenya.

## Supplementary Information

Supplementary Informations.

## Data Availability

The datasets generated and/or analysed during the current study are available from the corresponding author upon request and shall also be made available through open source platforms.
