# Supplementary material for: Mitogenomic analysis of diversity of key whitefly pests in Kenya and its implication to their sustainable management
Source: Sci Rep. 2021 Mar 18;11:6348. doi: 10.1038/s41598-021-85902-2 (PMC7973771; doi:10.1038/s41598-021-85902-2)
Supplement: Supplementary file 1 — Supplementary Informations. [file 41598_2021_85902_MOESM1_ESM.docx]

**Mitogenomic analysis of diversity of key whitefly pests in Kenya and its implication to their sustainable management**

Fathiya M. Khamis^1*^, Fidelis L. O. Ombura^1^, Inusa J. Ajene^1^, Komivi S. Akutse^1^, Sevgan Subramanian^1^, Samira A. Mohamed^1^, Thomas Dubois^1^, Chrysantus M. Tanga^1^ and Sunday Ekesi^1^

^1^Plant Health Theme, International Centre of Insect Physiology and Ecology (*icipe*), P.O. Box 30772-00100, Nairobi, Kenya

***Corresponding author:** [fkhamis@icipe.org](mailto:fkhamis@icipe.org)

**Supplementary Information**

**Supplementary Table 1**: Summary of the collection points of the whitefly samples assayed in this study

| **Village, Location** | **County** | **GPS coordinates** | **Elevation (m)** | **Host plant** | **Cultivation** |
| --- | --- | --- | --- | --- | --- |
| Mugusi, Keroka | Kisii | S00°40'41.8" E035°03'14.4" | 1838 | Tomato | Open field |
| Daiga, Nanyuki | Laikipia | N00°04'34.5" E037°07'07.2" | 1862 | Tomato | Open field |
| Timbila, Taveta | Taita Taveta | S03°22'53.3" E037°43'11.5" | 767 | Tomato | Green house |
| Jikaze, Nyahururu | Laikipia | S00°04'04.7" E036°32'06.9" | 2366 | Tomato | Green house |
| Scheme, Matuu | Machakos | S00°09'47.4" E037°31'52.6" | 1270 | French beans | Open field |
| Mbirikene, Meru | Meru | N00°00'21.6" E037°51'03.9" | 920 | Tomato | Open field |
| Teebere, Mwea East (Sub county) | Kirinyaga | S00°41'37.4" E037°22'12.2" | 1157 | Tomato | Open field |
| Sergoit | Elgeyo Marakwet | N00°41'18.3" E035°25'49.7" | 2206 | Tomato | Open field |
| Wote | Makueni | S01°39'40.8" E037°27'00.1" | 1881 | Tomato | Open field |
| Likuyani | Kakamega | N00°44'53.0" E035°07'00.3" | 1913 | Tomato | Green house |
| *icipe*, Nairobi | Nairobi | S01°13'26.6" E036°53'49.1" | 1608 | Tomato | Green house |
| Ngarariga, Limuru | Kiambu | S01°05'01.67" E036°37'30.59" | 2356 | Tomato | Open field |
| Kangubiri, Nyeri | Nyeri | S00°29'12.1" E037°01'33.8" | 1702 | Tomato | Open field |
| Ngamini, Ndaragwa | Nyandarua | S00°00'50.7" E036°35'02.2" | 2141 | French beans | Open field |
| Ngamini, Ndaragwa | Nyandarua | S00°01'19.2" E036°34'30.4" | 2156 | Tomato | Open field |
| Ihindu, Naivasha | Nakuru | S00°49'28.2" E036°32'43.2" | 2512 | French beans | Open field |
| Mihago,Subukia | Nakuru | N00°03'02.9" E036°13'42.6" | 1955 | French beans | Open field |
| Kiangochi, Murang'a | Murang'a | S00°45'20.9" E037°08'45.4" | 1292 | Cassava | Open field |
| Daiga, Nanyuki | Laikipia | N00°04'37.9" E037°07'10.0" | 1856 | French beans | Open field |
| Ngamini, Ndaragwa | Nyandarua | S00°00'52.6" E036°35'02.1" | 2141 | Kales | Open field |
| Borabu | Kisii | S00°46'54.8" E035°03'06.5" | 1878 | Tomato | Open field |
| Suna West | Migori | S01°03'47.0" E034°28'09.3" | 1358 | Tomato | Open field |
| Rangwe | HomaBay | S00°28'23.1" E034°33'45.5" | 1155 | Tomato | Open field |
| Cheptais | Bungoma | N00°48'47.1" E034°29'15.3" | 1671 | Tomato | Open field |
| Busia | Busia | N00°26'53.2" E034°06'38.5" | 1213 | Tomato | Open field |
| Mbironi, Loitoktok | Kajiado | S02°50'57.9" E037°32'15.9" | 1427 | Tomato | Open field |
| Moiben, Eldoret | Uasin Gichu | N00°52'21.4" E035°21'54.7" | 1940 | Tomato | Open field |
| Kinondo | Kwale | S04°19'27.1" E039°31'13.0" | 48 | Tomato | Open field |
| Takaungu | Kilifi | S03°42'05.1" E039°49'53.0" | 35 | Tomato | Open field |
| Marikebuni | Kilifi | S03°05'04.8" E040°06'14.5" | 46 | Tomato | Open field |
| Kamiti Prison | | S01°13.313 E036°53.729 | 1560 | Tomato | Open field |
| Ruturu |  | S01°09.247 E036°53.023 | 1580 | Tomato | Open field |
| Wiyumiririe, Ngobit | Laikipia | N00°04'09.9" E036°34'27.8" | 2236 | French beans | Open field |
| Ngamini, Ndaragua | Nyandarua | S00°00'52.6" E036°35'02.1" | 2141 | Kales | Open field |
| Giakanja | Nyeri | S00°27'59.8" E036°56'22.1" | 1860 | Kales | Open field |
| Giakanja | Nyeri | S00°27'28.50" E036°57'01.41" | 1804 | Pumpkin leaves | Open field |
| Mugoya, Embu | Embu | S00°32'24.2" E037°28'39.6" | 1359 | Tomato | Green house |
| Mihago, Subukia | Nakuru | N00°03'22.9" E036°13'44.7" | 1956 | Tomato | Open field |
| Olbolosat, Olkalou | Nyandarua | S00°11'31.78" E036°29'00.52" | 2375 | Kales | Open field |
| Olbolosat, OlKalou | Nyandarua | S00°11'47.7" E036°28'47.8" | 2356 | Pumpkin leaves | Open field |
| Narumoru | Nyeri | S00°13'17.93" E037°06'23.52" | 2171 | Kales | Open field |

**Supplementary Table 2**: Summary of regions where whiteflies were sighted during the survey in Kenya

| **Location** | **County** | **GPS coordinates** |
| --- | --- | --- |
| Leonard Mwauganga, Kighala village | Taita Taveta | S03°23'25.7" E038°20'13.8" |
| Sarah Mwakiriti, Kirema village | Taita Taveta | S03°23'00.8" E038°20'18.0" |
| Duncan Kariuki | Embu County | S00°28'59.5" E037°41'07.3" |
| Nicholas Njagi | Embu County | S00°30'26.2" E037°39'37.1" |
|  | Embu County | S00°30'49.0" E037°38'58.5" |
|  | Kathiani | S01°24.606° E037°19.971° |
| Site 1, Giakanja | Nyeri | S00°27'59.8" E036°56'22.1" |
| Site 2, Giakanja | Nyeri | S00°27'28.50" E036°57'01.41" |
| Site 3 (Mugoya), Embu | Embu | S00°32'24.2" E037°28'39.6" |
| Site 4 (Mihago), Subukia | Nakuru | N00°03'22.9" E036°13'44.7" |
| Site 5 (Olbolosat), Olkalou | Nyandarua | S00°11'31.78" E036°29'00.52" |
| Site 6 (Olbolosat), Olkalou | Nyandarua | S00°11'47.7" E036°28'47.8" |
| Site 7, Naromoru | Nyeri | S00°13'17.93" E037°06'23.52" |
| Borabu | Kisii | S00°46'54.8" E035°03'06.5" |
| Suna West | Migori | S01°03'47.0" E034°28'09.3" |
| Rangwe | HomaBay | S00°28'23.1" E034°33'45.5" |
| Cheptais | Bungoma | N00°48'47.1" E034°29'15.3" |
| Busia | Busia | N00°26'53.2" E034°06'38.5" |
| Mbironi | Kajiado | S02°50'57.9" E037°32'15.9" |
| Moiben | Uasin Gichu | N00°52'21.4" E035°21'54.7" |
| Kinondo | Kwale | S04°19'27.1" E039°31'13.0" |
| Takaungu | Kilifi | S03°42'05.1" E039°49'53.0" |
| Marikebuni | Kilifi | S03°05'04.8" E040°06'14.5" |
| Mpeketoni | Lamu | S02° 23'23.028'' E040°41'44.952'' |
| Mwea | Kirinyaga | S00°36'24.2" E037°22'27.0" |
| Gichugu | Kirinyaga | S00°27'52.7" E037°18'49.4" |
| Kisii | Kisii | S00°46'54.8" E035°03'06.5" |
| Taveta | Taita Taveta | S03°25'53.5" E037°43'57.6" |
| Loitoktok | Kajiado | S02°55'48.06" E037°30'27.91" |
| Isiolo | Isiolo | N00°19'45.64' E037°33'23.47" |
| Meru | Meru | N00°00'34.6° E037°51'35.6" |
| Nakuru | Nakuru | S00°09'05.8" E036°08'41.6" |
| Nyahururu | Laikipia | N00°03'20.4" E036°21'48.2" |
| Nyeri | Nyeri | S00°27'28.50" E036°57'01.41" |
| Kwale | Kwale | S04°19'27.1 E039°31'13.0" |
| Malindi | Kilifi | S03°08'36.0" E040°06'26.6" |
| Kilifi | Kilifi | S03°34'24.4" E039°49'10.4" |
| Murang'a | Murang'a | S00°45'08.6" E037°08'00.59" |
| Limuru | Kiambu | S01°04'07.89" E036°37'58.36" |
| Kiambu | Kiambu | S00°54'46.72 E037°04'51.8" |
| Machakos | Machakos | S01°10'01.5" E037°31'45.5" |
| Kitui | Kitui | S00°50"48.9" E038°00'23.6" |
| Thika | Kiambu | S01°03'41.80" E037°10'49.44" |
| Sergoit | Elgeyo-Marakwet | N00°41'17.86" E035°25'50.20" |
| Eldoret | Uasin Gishu | N00°52'21.4" E035°21'54.7" |
| Embu | Embu | S00°28'28.3" E037°34'53.0 |
| Migori | Migori | S01°05'16.05" E034°25'58.61" |
| Homa Bay | Homa Bay | S00°27'31.4" E034°38'08.6" |
| Kakamega | Kakamega | N00°40'45.5" E035°08'03.8" |
| Bungoma | Bungoma | N00°47'02.1" E034°30'48.9" |
| Nanyuki | Laikipia | N00°04'07.5" E037°06'45.2" |
| Makueni | Makueni | S00°23'00.51" E038°00'16.79" |
| Kilgoris | Narok | S00°54'56.31" E034°57'07.95" |
| Narok | Narok | S00°06'13.34" E035°51'55.84" |
| OlKalou | Nyandarua | S00°11'47.9" E036°28'48.5" |
| Wiyumirie | Laikipia | S00°04'04.7" E036°32'06.9" |
| Lamu | Lamu | S02°23'09.83" E040°26'58.23" |
| Trans Nzoia | Trans Nzoia | N00°58'59.73" E035°05'53.39" |
| Kisii | Kisii | S00°45'57.1" E034°59'22.3" |
| Taveta | Taita Taveta | S03°25'53.5" E037°43'57.6" |
| Mwatate | Taita Taveta | S03°33'32.68" E038°22'39.48" |
| Nyahururu | Laikipia | N00°03'13.8" E036°21'51.3" |
| Kilifi | Kilifi | S03°41'11.45" E039°51'21.16" |
| Kabete | Kiambu | S01°14'28.66" E036°44'47.80" |
| Limuru | Kiambu | S01°05'21.12" E036°38'37.89" |
| Machakos | Machakos | S01°06'52.39" E037°22'24.52" |
| Migori | Migori | S01°03'30.9" E034°27'56.1" |
| Kakamega | Kakamega | N00°42'05.4" E035°09'15.1" |
| Busia | Busia | N00°24'55.66" E034°06'18.36" |
| Nairobi | Nairobi | S01°16'21.51" E036°43'06.41" |
| Naivasha | Nakuru | S00°37'35.66" E036°22'49.47" |
| Subukia | Nakuru | N00°03'02.9" E036°13'42.6" |
| Bahati | Nakuru | S00°09'05.8" E036°08'41.6" |
| OlKalou | OlKalou | S00°11'47.9" E036°28'48.5" |
| Nanyuki | Laikipia | N00°04'39.9" E037°07'00.0" |
| Nyeri | Nyeri | S00°13'26.6" E037°03'08.9" |
| Nyeri | Nyeri | S00°29'55.0" E037°01'08.3" |
| Embu | Embu | S00°28'16.0" E037°34'53.0" |
| Meru | Meru | S00°08'23.0" E037°47'50.8" |
| Nanyuki | Laikipia | N00°04'33.6" E037°07'17.4" |
| Limuru | Kiambu | S01°05'01.3" E036°37'29.1" |
| Siaya | Siaya | S00°12'36.3" E034°19'23.4" |
| Homa Bay | Homa Bay | S00°27'59.5" E034°36'32.6" |
| Embu | Embu | S00°48'37.6" E037°50'10.8" |
| Meru | Meru | S00°00'57.2" E037°46'24.1" |
| Migori | Migori | S01°06'21.2" E034°28'18.2" |
| Homa Bay | Homa Bay | S00°23'00.3" E034°39'08.5" |
| Siaya | Siaya | S00°12'36.3" E034°19'23.4" |
| Meru | Meru | N00°00'21.6" E037°51'03.9" |
| Embu | Embu | S00°32'33.9" E037°28'20.8" |
| Migori | Migori | S01°03'47.0" E034°28'09.3" |
| Homa Bay | Homa Bay | S00°28'23.1" E034°33'45.5" |
| Siaya | Siaya | S00°06'03.3" E034°16'48.1" |

**Supplementary Table 3**: Primers used in PCR amplification and bidirectional sequencing of the 16S ribosomal RNA region in Whiteflies for species identification

| **Name** | **Sequence5' - 3'** | **Target** | **Source** | **Annealing Temp (°C)** |
| --- | --- | --- | --- | --- |
| White Fw | CCGGTTTGAACTCAGATCATGT | 16S rDNA | Simon *et al*., 1994; Frohlich *et al*., 1999 | 57.4 |
| White Rv | CGCCTGTTTAACAAAAACAT | 16S rDNA |  |  |
| 4119 Fw | CGCCTGTTTAACAAAAACAT | 16S rDNA | Xiong and Kocher, 1991 | 55.5 |
| 4118 Rv | CCGGTCTGAACTCAGATCACGT | 16S rDNA |  |  |
| LCO1490 | GGTCAACAAATCATAAAGATATTGG | COI | Folmer *et al*., 1994 | 50.6 |
| HCO2198 | TAAACTTCAGGGTGACCAAAAAATA | COI |  |  |

**Supplementary Table 4**: Summary of samples used in the phylogenetic analysis of whitefly species along with publicly available sequences from GenBank

| **4119 FW/4118 Rv** | **White FW/ White RV** |  |
| --- | --- | --- |
| Aleurodicus dispersus (JQ305694) | Aleurodicus dispersus (KR063274) |  |
| Aleyrodes proletella (GQ867758) | Aleyrodes proletella (GQ867758) |  |
| Aphis gossypii (NC_024581) | Aphis gossypii (NC_024581) |  |
| Bemisia afer (GQ867747) | Bemisia afer (GQ867747) |  |
| Bemisia tabaci (JQ906700) | Bemisia tabaci (KJ778614) |  |
| Embu-1-ex-butternut | Embu-1-ex-butternut |  |
| Embu-1-ex-pumpkin | Embu-1-ex-pumpkin |  |
| Embu-1-ex-tomato | Embu-1-ex-Tomato-white |  |
| Embu-2-ex-butternut | Embu-2-ex-butternut |  |
| Embu-2-ex-pumpkin | Embu-2-ex-pumpkin |  |
| Embu-2-ex-tomato | Embu-2-ex-Tomato-white |  |
| Embu-3-ex-butternut | Embu-3-ex-butternut |  |
| Embu-3-ex-pumpkin | Embu-3-ex-pumpkin |  |
| Embu-3-ex-tomato | Embu-3-ex-Tomato-white |  |
| Embu-4-ex-butternut | Embu-4-ex-butternut |  |
| Embu-4-ex-pumpkin | Embu-4-ex-pumpkin |  |
| Embu-4-ex-tomato | Embu-4-ex-Tomato-white |  |
| Embu-5-ex-butternut | Embu-5-ex-butternut |  |
| Embu-5-ex-pumpkin | Embu-5-ex-pumpkin |  |
| Embu-5-ex-tomato | Embu-5-ex-Tomato-white |  |
| Embu-6-ex-butternut | Embu-6-ex-butternut |  |
| Embu-6-ex-pumpkin | Embu-6-ex-pumpkin |  |
| Embu-6-ex-tomato | Embu-6-ex-Tomato-white |  |
| Embu-7-ex-butternut | Embu-7-ex-butternut |  |
| Embu-7-ex-pumpkin | Embu-7-ex-pumpkin |  |
| Embu-7-ex-tomato | Embu-7-ex-Tomato-white |  |
| ex-tomato-1 | Kamiti-1 |  |
| ex-tomato-2 | Kathiani-1 |  |
| ex-tomato-3 | Kathiani-2 |  |
| ex-tomato-4 | Kathiani-3 |  |
| ex-tomato-5 | Kathiani-4 |  |
| ex-tomato-6 | Kathiani-5 |  |
| ex-tomato-7 | Kathiani-6 |  |
| Icipe-1-4119 | Kathiani-7 |  |
| Icipe-2-4119 | Limuru-2 |  |
| Icipe-3-4119 | Limuru-3 |  |
| Icipe-4-4119 | Limuru-5 |  |
| Icipe-5-4119 | Limuru-6 |  |
| Kakamega-1-4119 | Muranga-1-CA |  |
| Kamiti-1 | Muranga-1-CB |  |
| Kamiti-2 | Muranga-2-CA |  |
| Kathiani-1 | Muranga-2-CB |  |
| Kathiani-2 | Muranga-3-CA |  |
| Kathiani-3 | Muranga-3-CB |  |
| Kathiani-4 | Muranga-4-CA |  |
| Kathiani-5 | Muranga-5-CA |  |
| Kathiani-6 | Muranga-5-CB |  |
| Kathiani-7 | Naivasha-1 |  |
| Kisii1-4118 | Naivasha-2 |  |
| Kisii2-4118 | Naivasha-3 |  |
| Kisii3-4118 | Naivasha-4 |  |
| Kisii4-4118 | Naivasha-5 |  |
| Kisii5-4118 | Nanyuki-1-FB |  |
| Kisii6-4118 | Nanyuki-2-FB |  |
| Kisii7-4118 | Nanyuki-4-FB |  |
| Limuru-1-4119 | Nanyuki-5-FB |  |
| Limuru-2-4119 | Narumoru-1-ex-kales |  |
| Limuru-3-4119 | Narumoru-2-ex-kales |  |
| Limuru-4-4119 | Narumoru-3-ex-kales |  |
| Limuru-5-4119 | Ndaragua-1-FB |  |
| Matuu2-4118 | Ndaragua-1-KL |  |
| Matuu3-4118 | Ndaragua-2 |  |
| Matuu4-4118 | Ndaragua-2-FB |  |
| Matuu5-4118 | Ndaragua-3 |  |
| Meru1-4118 | Ndaragua-3-KL |  |
| Meru3-4118 | Ndaragua-4 |  |
| Meru6-4118 | Ndaragua-4-FB |  |
| Meru7-4118 | Ndaragua-4-KL |  |
| Muranga-CA-1-4119 | Ndaragua-5 |  |
| Muranga-CA-3-4119 | Ndaragua-5-FB |  |
| Muranga-CA-4-4119 | Ndaragua-5-KL |  |
| Muranga-CA-5-4119 | Nyeri-1 |  |
| Muranga-CB-1-4119 | Nyeri-1-ex-kales-white |  |
| Muranga-CB-3-4119 | Nyeri-1-ex-pumpkin-white |  |
| Muranga-CB-4-4119 | Nyeri-2 |  |
| Muranga-CB-5-4119 | Nyeri-2-ex-kales-white |  |
| Naivasha-1-4119 | Nyeri-2-ex-pumpkin-white |  |
| Naivasha-2-4119 | Nyeri-3 |  |
| Naivasha-3-4119 | Nyeri-3-ex-kales-white |  |
| Naivasha-5-4119 | Nyeri-3-ex-pumpkin-white |  |
| Nanyuki1-4118 | Nyeri-4 |  |
| Nanyuki2-4118 | Nyeri-4-ex-kales-white |  |
| Nanyuki3-4118 | Nyeri-4-ex-pumpkin-white |  |
| Nanyuki4-4118 | Nyeri-5 |  |
| Nanyuki5-4118 | Nyeri-5-ex-pumpkin-white |  |
| Nanyuki6-4118 | Nyeri-6-ex-kales-white |  |
| Nanyuki7-4118 | Nyeri-6-ex-pumpkin-white |  |
| Nanyuki-F-1-4119 | Nyeri-7-ex-kales-white |  |
| Nanyuki-F-2-4119 | Nyeri-7-ex-pumpkin-white |  |
| Nanyuki-F-4-4119 | Olkalou-1-ex-kales-white |  |
| Narumoru-1-ex-kales | Olkalou-1-ex-pumpkin-white |  |
| Narumoru-2-ex-kales | Olkalou-2-ex-kales-white |  |
| Narumoru-3-ex-kales | Olkalou-2-ex-pumpkin-white |  |
| Narumoru-5-ex-kales | Olkalou-3-ex-kales-white |  |
| Narumoru-6-ex-kales | Olkalou-3-ex-pumpkin-white |  |
| Narumoru-7-ex-kales | Olkalou-4-ex-kales-white |  |
| Ndaragua-1-4119 | Olkalou-4-ex-pumpkin-white |  |
| Ndaragua-2-4119 | Olkalou-5-ex-kales-white |  |
| Ndaragua-3-4119 | Olkalou-5-ex-pumpkin-white |  |
| Ndaragua-4-4119 | Olkalou-6-ex-kales-white |  |
| Ndaragua-5-4119 | Olkalou-6-ex-pumpkin-white |  |
| Ndaragua-F-1-4119 | Olkalou-7-ex-kales-white |  |
| Ndaragua-F-2-4119 | Olkalou-7-ex-pumpkin-white |  |
| Ndaragua-F-3-4119 | Ruturu-1 |  |
| Ndaragua-F-4-4119 | Ruturu-2 |  |
| Ndaragua-F-5-4119 | Ruturu-3 |  |
| Ndaragua-K-1-4119 | Ruturu-4 |  |
| Ndaragua-K-3-4119 | Ruturu-5 |  |
| Ndaragua-K-4-4119 | Ruturu-6 |  |
| Ndaragua-K-5-4119 | Subukia-1-ex-tomato-white |  |
| Nyahururu-1-4118 | Subukia-1-FB |  |
| Nyahururu-2-4118 | Subukia-2-ex-tomato-white |  |
| Nyahururu-3-4118 | Subukia-2-FB |  |
| Nyahururu-4-4118 | Subukia-3-ex-tomato-white |  |
| Nyahururu-5-4118 | Subukia-3-FB |  |
| Nyahururu-6-4118 | Subukia-4-ex-tomato-white |  |
| Nyahururu-7-4118 | Subukia-5-ex-tomato-white |  |
| Nyeri-1-4119 | Subukia-6-ex-tomato-white |  |
| Nyeri-1-ex-kales | Subukia-7-ex-tomato-white |  |
| Nyeri-1-ex-pumpkin | Tomato-1 |  |
| Nyeri-2-4119 | Tomato-2 |  |
| Nyeri-2-ex-kales | Tomato-3 |  |
| Nyeri-2-ex-pumpkin | Tomato-4 |  |
| Nyeri-3-4119 | Tomato-5 |  |
| Nyeri-3-ex-kales | Tomato-6 |  |
| Nyeri-3-ex-pumpkin | Tomato-7 |  |
| Nyeri-4-4119 | Trialeurodes vaporariorum (JF693942) |  |
| Nyeri-4-ex-kales | Werugha-1 |  |
| Nyeri-4-ex-pumpkin | Werugha-2 |  |
| Nyeri-5-4119 | W-Kisii-1 |  |
| Nyeri-5-ex-kales | W-Kisii-2 |  |
| Nyeri-5-ex-pumpkin | W-Kisii-3 |  |
| Nyeri-6-ex-kales | W-Kisii-4 |  |
| Nyeri-6-ex-pumpkin | W-Kisii-5 |  |
| Nyeri-7-ex-kales | W-Kisii-6 |  |
| Nyeri-7-ex-pumpkin | W-Kisii-7 |  |
| Olkalou-1-ex-kales | W-Matuu-1 |  |
| Olkalou-1-ex-pumpkin | W-Matuu-3 |  |
| Olkalou-2-ex-kales | W-Matuu-5 |  |
| Olkalou-2-ex-pumpkin | W-Matuu-6 |  |
| Olkalou-3-ex-kales | W-Matuu-7 |  |
| Olkalou-3-ex-pumpkin | W-Meru-1 |  |
| Olkalou-4-ex-kales | W-Meru-3 |  |
| Olkalou-4-ex-pumpkin | W-Meru-5 |  |
| Olkalou-5-ex-kales | W-Meru-6 |  |
| Olkalou-5-ex-pumpkin | W-Meru-7 |  |
| Olkalou-6-ex-kales | W-Nanyuki-1 |  |
| Olkalou-6-ex-pumpkin | W-Nanyuki-2 |  |
| Olkalou-7-ex-kales | W-Nanyuki-3 |  |
| Olkalou-7-ex-pumpkin | W-Nanyuki-4 |  |
| Ruturu-1 | W-Nanyuki-5 |  |
| Ruturu-2 | W-Nanyuki-6 |  |
| Ruturu-3 | W-Nyahururu-1 |  |
| Ruturu-4 | W-Nyahururu-2 |  |
| Ruturu-5 | W-Nyahururu-3 |  |
| Ruturu-6 | W-Nyahururu-4 |  |
| Sergoit-1-4119 | W-Nyahururu-5 |  |
| Sergoit-2-4119 | W-Nyahururu-6 |  |
| Sergoit-3-4119 | W-Nyahururu-7 |  |
| Sergoit-4-4119 | W-Taveta-1 |  |
| Sergoit-5-4119 | W-Taveta-2 |  |
| Subukia-1-ex-tomato | W-Taveta-3 |  |
| Subukia-2-ex-tomato | W-Taveta-4 |  |
| Subukia-3-ex-tomato | W-Taveta-5 |  |
| Subukia-4-ex-tomato | W-Taveta-6 |  |
| Subukia-5-ex-tomato | W-Taveta-7 |  |
| Subukia-6-ex-tomato | W-Teebere-1 |  |
| Subukia-7-ex-tomato | W-Teebere-2 |  |
| Subukia-F-1-4119 | W-Teebere-3 |  |
| Subukia-F-2-4119 | W-Teebere-4 |  |
| Subukia-F-3-4119 | W-Teebere-5 |  |
| Subukia-F-5-4119 | W-Teebere-6 |  |
| Taveta1-4118 | W-Teebere-7 |  |
| Taveta2-4118 |  |  |
| Taveta3-4118 |  |  |
| Taveta4-4118 |  |  |
| Taveta5-4118 |  |  |
| Taveta6-4118 |  |  |
| Taveta7-4118 |  |  |
| Teebere1-4118 |  |  |
| Teebere2-4118 |  |  |
| Teebere3-4118 |  |  |
| Teebere4-4118 |  |  |
| Teebere5-4118 |  |  |
| Teebere6-4118 |  |  |
| Teebere7-4118 |  |  |
| Trialeurodes vaporariorum (GQ867761) |  | |
| Werugha-1 |  |  |
| Werugha-2 |  |  |
| Wote-1-4119 |  |  |
| Wote-2-4119 |  |  |
| Wote-3-4119 |  |  |
| Wote-4-4119 |  |  |
| Wote-5-4119 |  |  |

**Supplementary Table 5.** List of the eight mitochondrial sequences used in the phylogenetic reconstruction of the family Aleyrodidae with common name, GenBank accession numbers and references. *Aphis gossypii* (Hemiptera: Aphididae) was used as an outgroup.

| **Species** | **Common name** | **GenBank** | **Reference** | **Size (bp)** | **Status** |
| --- | --- | --- | --- | --- | --- |
| *Aleurodicus dispersus* | Spiralling Whitefly | KR063274 | Ming-Xing *et al*. 2015 | 16170 | Partial |
| *Aleurocanthus spiniferus* | Spiny Whitefly | NC_029155 | Chen *et al*. 2016 | 15220 | Complete |
| *Aleurodicus dugesii* | Doogie Howzer Whitefly | NC_005939 | Ming-Xing *et al*. 2017 | 15723 | Complete |
| *Aleurochiton aceris* | NA | NC_006160 | Wang *et al*. 2019 | 15388 | Complete |
| *Aphis gossypii* | Aphid | NC_024581 | Zhang *et al*. 2014 | 15869 | Complete |
| *Bemisia afer* | Cotton Whitefly | KF734668 | Wang *et al*. 2013 | 14968 | Complete |
| *Bemisia tabaci* | Silverleaf Whitefly | KJ778614 | Tay *et al*. 2014 | 15632 | Complete |
| *Trialeurodes vaporariorum* | Greenhouse Whitefly | NC_006280 | Thao *et al*. 2003 | 18414 | Complete |
| *Tetraleurodes acaciae* | Acacia Whitefly | AY521262 | Chen *et al*. 2016 | 15080 | Complete |

**Supplementary Table 6a**. Gene composition and order of the complete mitochondrial genome of *Aleurodicus dispersus*.

|  |  | **Sample 4: *Aleurodicus dispersus*** | | | |
| --- | --- | --- | --- | --- | --- |
| Name | Type | Minimum | Maximum | Length | Direction |
| COX1 CDS | CDS | 1 | 1,550 | 1,550 | forward |
| tRNA-Leu | tRNA | 1,551 | 1,616 | 66 | forward |
| COX2 CDS | CDS | 1,617 | 2,277 | 661 | forward |
| tRNA-Lys | tRNA | 2,278 | 2,347 | 70 | forward |
| tRNA-Asp | tRNA | 2,359 | 2,421 | 63 | forward |
| ATP8 CDS | CDS | 2,422 | 2,571 | 150 | forward |
| ATP6 CDS | CDS | 2,567 | 3,217 | 651 | forward |
| COX3 CDS | CDS | 3,218 | 4,013 | 796 | forward |
| tRNA-Gly | tRNA | 4,013 | 4,073 | 61 | forward |
| ND3 CDS | CDS | 4,074 | 4,433 | 360 | forward |
| tRNA-Ala | tRNA | 4,437 | 4,499 | 63 | forward |
| tRNA-Arg | tRNA | 4,500 | 4,564 | 65 | forward |
| 12S rRNA | rRNA | 5,473 | 6,208 | 736 | forward |
| tRNA-Val | tRNA | 6,206 | 6,271 | 66 | forward |
| 16S rRNA | rRNA | 6,272 | 7,469 | 1,198 | forward |
| tRNA-Leu | tRNA | 7,486 | 7,554 | 69 | forward |
| ND1 CDS | CDS | 7,557 | 8,513 | 957 | reverse |
| ND6 CDS | CDS | 9,240 | 9,899 | 660 | forward |
| CYTB CDS | CDS | 9,907 | 11,021 | 1,115 | forward |
| tRNA-Ser | tRNA | 11,020 | 11,077 | 58 | reverse |
| tRNA-Asn | tRNA | 11,080 | 11,136 | 57 | forward |
| tRNA-Glu | tRNA | 11,140 | 11,204 | 65 | forward |
| tRNA-Phe | tRNA | 11,193 | 11,258 | 66 | reverse |
| ND5 CDS | CDS | 11,260 | 12,928 | 1,669 | reverse |
| tRNA-His | tRNA | 12,929 | 12,995 | 67 | reverse |
| ND4 CDS | CDS | 12,992 | 14,271 | 1,280 | reverse |
| ND4L CDS | CDS | 14,267 | 14,571 | 305 | reverse |
| tRNA-Thr | tRNA | 14,573 | 14,639 | 67 | forward |
| tRNA-Pro | tRNA | 14,638 | 14,700 | 63 | reverse |
| tRNA-Ile | tRNA | 14,705 | 14,784 | 80 | forward |
| tRNA-Met | tRNA | 14,785 | 14,851 | 67 | forward |
| ND2 CDS | CDS | 14,870 | 15,839 | 970 | forward |
| tRNA-Trp | tRNA | 15,825 | 15,890 | 66 | forward |
| tRNA-Tyr | tRNA | 15,889 | 15,955 | 67 | reverse |
| tRNA-Cys | tRNA | 15,951 | 16,013 | 63 | forward |

**Supplementary Table 6b.** Gene composition and order of the complete mitochondrial genome of *Bemisia afer*

|  |  | **Sample 3: *Bemisia afer*** | | | |
| --- | --- | --- | --- | --- | --- |
| Name | Type | Minimum | Maximum | Length | Direction |
| COX1 gene | gene | 1 | 1537 | 1537 | forward |
| tRNA-Leu | trna | 1,538 | 1,604 | 67 | forward |
| COX2 gene | gene | 1,605 | 2,271 | 667 | forward |
| tRNA-Lys | trna | 2,272 | 2,341 | 70 | forward |
| ATP8 gene | gene | 2,365 | 2,577 | 213 | forward |
| ATP6 gene | gene | 2,584 | 3,234 | 651 | forward |
| tRNA-Ser | trna | 3,268 | 3,324 | 57 | reverse |
| tRNA-Glu | trna | 3,325 | 3,391 | 67 | reverse |
| tRNA-Phe | trna | 3,392 | 3,457 | 66 | reverse |
| ND5 gene | gene | 3,458 | 5,105 | 1,648 | reverse |
| tRNA-His | trna | 5,109 | 5,174 | 66 | reverse |
| ND4 gene | gene | 5,181 | 6,485 | 1,305 | reverse |
| ND4L gene | gene | 6,464 | 6,748 | 285 | reverse |
| tRNA-Thr | trna | 6,756 | 6,818 | 63 | forward |
| tRNA-Pro | trna | 6,819 | 6,884 | 66 | reverse |
| ND6 gene | gene | 6,886 | 7,371 | 486 | forward |
| CYTB gene | gene | 7,371 | 8,501 | 1,131 | forward |
| tRNA-Ser | trna | 8,502 | 8,584 | 83 | forward |
| ND1 gene | gene | 8,585 | 9,491 | 907 | reverse |
| tRNA-Leu | trna | 9,492 | 9,557 | 66 | reverse |
| l-rRNA | rrna | 9,562 | 10,757 | 1,196 | reverse |
| tRNA-Val | trna | 10,758 | 10,819 | 62 | reverse |
| tRNA-Asp | trna | 10,822 | 10,893 | 72 | reverse |
| tRNA-Gln | trna | 10,906 | 10,970 | 65 | reverse |
| s-rRNA | rrna | 10,971 | 11,885 | 915 | reverse |
| tRNA-Asn | trna | 11,886 | 11,951 | 66 | reverse |
| tRNA-Arg | trna | 11,954 | 12,018 | 65 | reverse |
| tRNA-Ala | trna | 12,020 | 12,079 | 60 | reverse |
| ND3 gene | gene | 12,079 | 12,426 | 348 | reverse |
| tRNA-Gly | trna | 12,434 | 12,497 | 64 | reverse |
| COX3 gene | gene | 12,519 | 13,301 | 783 | reverse |
| tRNA-Ile | trna | 13,668 | 13,735 | 68 | forward |
| tRNA-Met | trna | 13,734 | 13,803 | 70 | forward |
| ND2 gene | gene | 13,804 | 14,772 | 969 | forward |
| tRNA-Trp | trna | 14,773 | 14,834 | 62 | forward |
| tRNA-Tyr | trna | 14,833 | 14,900 | 68 | reverse |
| tRNA-Cys | trna | 14,902 | 14,967 | 66 | reverse |

**Supplementary Table 6c.** Gene composition and order of the complete mitochondrial genome of *Trialeurodes vaporariorum*

|  |  | **Sample 2: *Trialeurodes vaporariorum*** | | | |
| --- | --- | --- | --- | --- | --- |
| Gene | Type | Minimum | Maximum | Length | Direction |
| COX1 gene | gene | 1 | 1,536 | 1,536 | forward |
| tRNA-Leu | trna | 1,546 | 1,610 | 65 | forward |
| COX2 gene | gene | 1,611 | 2,274 | 664 | forward |
| tRNA-Lys | trna | 2,275 | 2,344 | 70 | forward |
| tRNA-Asp | trna | 2,346 | 2,406 | 61 | forward |
| ATP8 gene | gene | 2,416 | 2,568 | 153 | forward |
| ATP6 gene | gene | 2,570 | 3,223 | 654 | forward |
| COX3 gene | gene | 3,259 | 4,047 | 789 | forward |
| ND3 gene | gene | 4,097 | 4,450 | 354 | forward |
| tRNA-Ala | trna | 4,482 | 4,542 | 61 | forward |
| tRNA-Arg | trna | 4,551 | 4,617 | 67 | forward |
| tRNA-Asn | trna | 4,618 | 4,682 | 65 | forward |
| tRNA-Ser | trna | 4,681 | 4,735 | 55 | forward |
| tRNA-Glu | trna | 4,731 | 4,795 | 65 | forward |
| tRNA-Phe | trna | 4,784 | 4,853 | 70 | reverse |
| ND5 gene | gene | 4,855 | 6,537 | 1,683 | reverse |
| tRNA-His | trna | 6,535 | 6,594 | 60 | reverse |
| ND4 gene | gene | 6,596 | 7,873 | 1,278 | reverse |
| ND4L gene | gene | 7,870 | 8,163 | 294 | reverse |
| tRNA-Thr | trna | 8,165 | 8,226 | 62 | forward |
| tRNA-Pro | trna | 8,225 | 8,287 | 63 | forward |
| ND6 gene | gene | 8,331 | 8,730 | 400 | forward |
| CYTB gene | gene | 8,790 | 9,920 | 1,131 | forward |
| tRNA-Ser | trna | 9,921 | 9,989 | 69 | forward |
| ND1 gene | gene | 9,987 | 10,919 | 933 | reverse |
| tRNA-Leu | trna | 10,917 | 10,982 | 66 | reverse |
| l-rRNA | rrna | 10,987 | 12,196 | 1,210 | reverse |
| tRNA-Val | trna | 12,197 | 12,260 | 64 | reverse |
| s-rRNA | rrna | 12,260 | 13,003 | 744 | reverse |
| tRNA-Gln | trna | 16,733 | 16,799 | 67 | reverse |
| tRNA-Ile | trna | 16,806 | 16,868 | 63 | forward |
| tRNA-Met | trna | 16,868 | 16,934 | 67 | forward |
| tRNA-Trp | trna | 17,915 | 17,981 | 67 | forward |
| tRNA-Gly | trna | 18,073 | 18,136 | 64 | forward |
| tRNA-Tyr | trna | 18,273 | 18,338 | 66 | reverse |
| tRNA-Cys | trna | 18,340 | 18,406 | 67 | reverse |
| ND2 gene | gene | <16944 | 17,906 | >963 | forward |

**Supplementary Table 7.** Pairwise genetic identities of 13 protein coding gene sequences from the complete mitochondrial genome of members of the Aleyrodidae family available in GenBank with *Aphis gossypii*

|  | 1 | 2 | 3 | 4 | 5 | 6 | 7 | 8 | 9 |
| --- | --- | --- | --- | --- | --- | --- | --- | --- | --- |
| *Aleurodicus_dispersus* (KR063274) | - | 48.55 | 45.12 | 49.75 | 47.79 | 46.73 | 45.09 | 94.01 | 47.8 |
| *Aphis gossypii* (NC_024581) | 48.55 | - | 45.07 | 49.7 | 34.36 | 46.76 | 45.04 | 49.1 | 48.93 |
| *Bemisia afer* (KF734668) | 45.12 | 45.07 | - | 64.49 | 34.75 | 48.5 | 99.56 | 45.5 | 51.49 |
| *Bemisia tabaci* (KJ778614) | 49.75 | 49.7 | 64.49 | - | 37.23 | 50.66 | 64.52 | 50.17 | 53.84 |
| *Aleyrodes proletella* (this study) | 47.79 | 34.36 | 34.75 | 37.23 | - | 32.18 | 34.8 | 48.36 | 33.36 |
| *Trialeurodes vaporariorum* (this study) | 46.73 | 46.76 | 48.5 | 50.66 | 32.18 | - | 48.48 | 47.16 | 94.52 |
| *Bemisia afer* (this study) | 45.09 | 45.04 | 99.56 | 64.52 | 34.8 | 48.48 | - | 45.48 | 51.47 |
| *Aleurodicus dispersus* (this study) | 94.01 | 49.1 | 45.5 | 50.17 | 48.36 | 47.16 | 45.48 | - | 48.27 |
| *Trialeurodes vaporariorum* (NC_006280) | 47.8 | 48.93 | 51.49 | 53.84 | 33.36 | 94.52 | 51.47 | 48.27 | - |


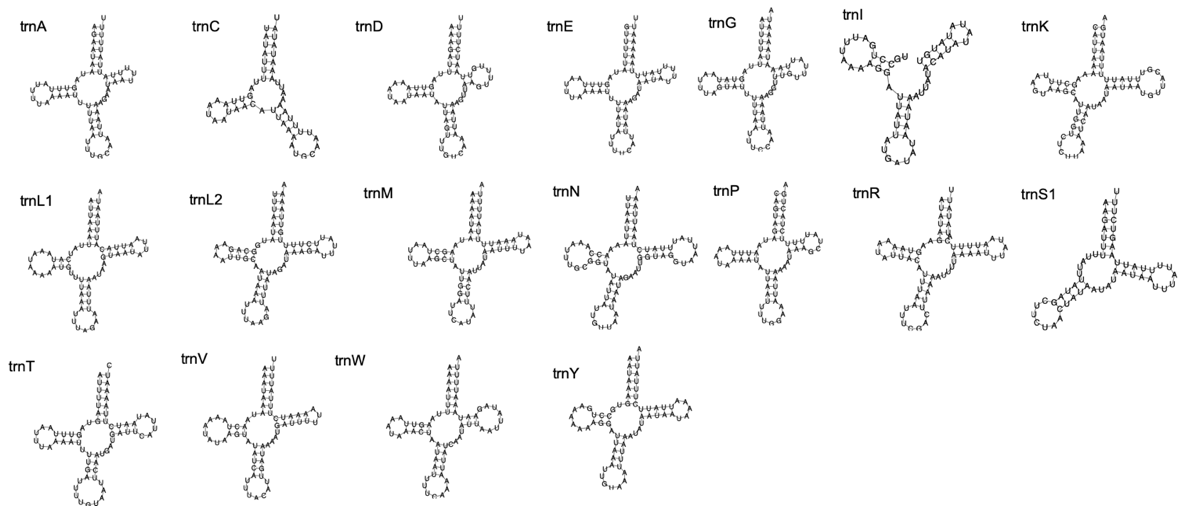


**Supplementary Figure 1**: Transfer RNA secondary structures of 18 tRNAs in *Aleurodicus dispersus* as predicted by MITOS.


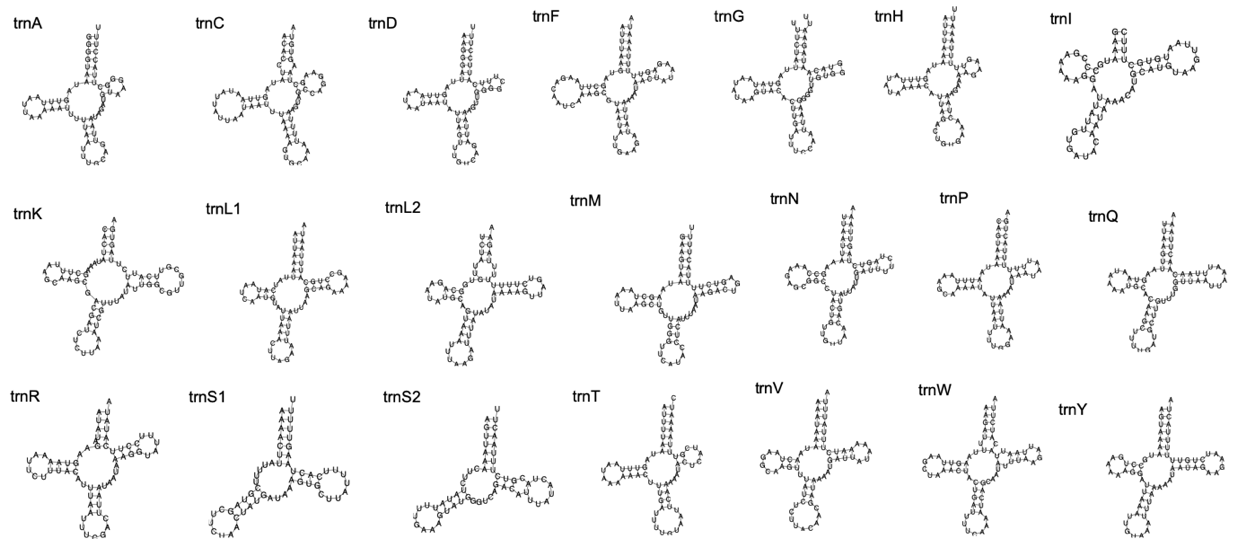


**Supplementary Figure 2**: Transfer RNA secondary structures of 21 tRNAs in *Trialeurodes vaporariorum* as predicted by MITOS.


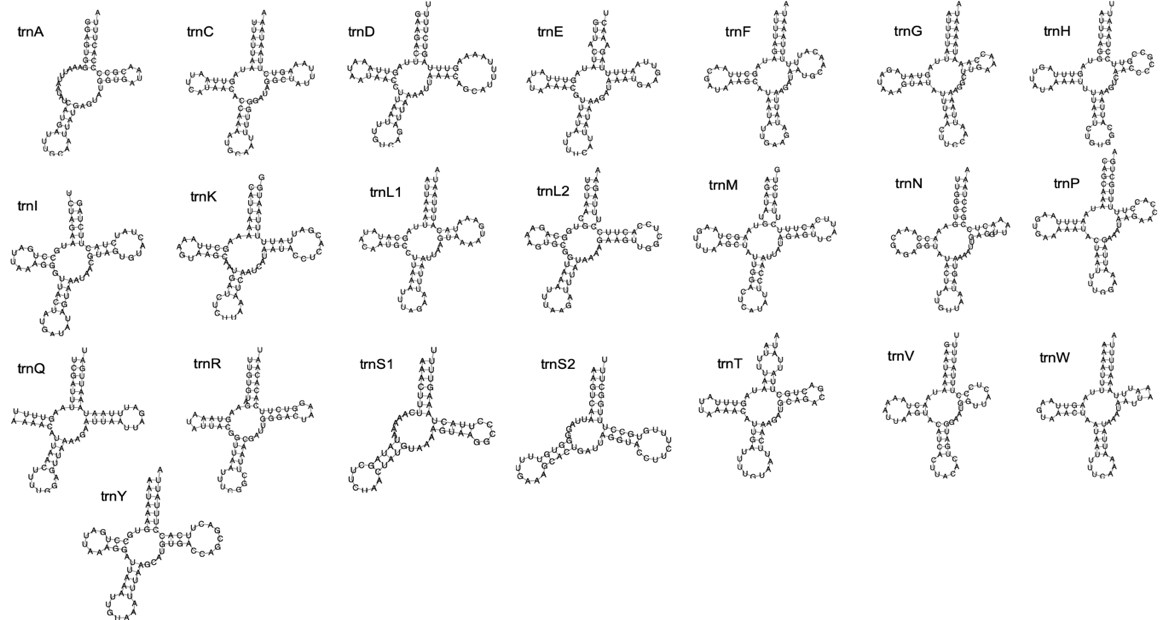


**Supplementary Figure 3** Transfer RNA secondary structures of 22 tRNAs in *Bemisia afer* as predicted by MITOS.
